# COVID-19 information seeking pattern and perceived benefits in Nigeria: a cross-sectional survey

**DOI:** 10.11604/pamj.2022.42.121.33350

**Published:** 2022-06-15

**Authors:** Lovelyn Ndubuisi-Okoroezi, Jennifer Ikechukwu-Okoroezi, Linda Odikpo, Chinenye Ifeoma Ubah, Chisom Joy Mbadugha

**Affiliations:** 1 National Health Service (NHS) Foundation Trust, Kent Hospitals University, Kent, United Kingdom; 2 Department of Nursing Sciences, Abia State University, Abia, Nigeria,; 3 Department of Nursing Sciences, Nnamdi Azikiwe University, Awka, Nigeria,; 4 Anglia Ruskin University Cambridge, Anglia, United Kingdom,; 5 Department of Nursing Sciences, University of Nigeria Enugu Campus, Enugu, Nigeria

**Keywords:** COVID-19, Nigeria, adults, pattern, perceived benefits

## Abstract

**Introduction:**

access to COVID-19 related information is fundamental to making accurate decisions and performing evidence-based actions aimed to control its spread and improve health. This study assessed pattern and perceived benefits of COVID-19 related information in Nigeria.

**Methods:**

a descriptive cross-sectional survey was carried out among adult Nigerian residents aged 18-years and above in the geopolitical zones using multistage sampling strategy. Questionnaire developed by World Health Organization (WHO) was adapted and pretested. Descriptive statistics were carried on the data and presented in frequencies, percentages, mean and standard deviation. Inferential statistics (Chi-square) was used to test differences between some categorical variables. Data obtained were analysed using statistical package for the social sciences (SPSS) software version 22.

**Results:**

from responses, 498 (47.9%) of participants highly desired information on COVID-19, while 311 (29.9%) frequently sought COVID-19 information. Majority 647 (62.3%) were interested in getting information on treatment protocols/home remedies for COVID-19 prevention. Also, 934 (89.9%) found information on COVID-19 beneficial. A significant difference existed between male and female respondents regarding government guidelines on public interaction (p=0.026) and home remedies to prevent COVID-19 infection (p=0.013). Consistently, more urban residents than rural sought information on majority of the COVID-19 related information.

**Conclusion:**

information on COVID-19 is highly sought by Nigerian residents and such should be widely disseminated regularly through authentic and verified social media channels. Nigerians are highly desirous of information on treatment protocols and home remedies to prevent COVID-19. Adequate provision of accurate and timely information by authorities through trusted sources will improve health literacy and help curb the spread of COVID-19.

## Introduction

The novel COVID-19 virus is a pandemic that posed serious threats to global stability and dwindling health systems, especially in developing countries like Nigeria which has recorded 214, 513 cases confirmed, 207,403 cases discharged and 2,980 deaths in its 36 states and the Federal Capital Territory [1]. With the emergence of COVID-19, the need for information sharing became a necessity across the globe including countries like Nigeria which WHO categorized as one of the 13 high-risk African countries concerning the spread of COVID-19 and equally among the vulnerable African nations, given the weak state of the healthcare system [2,3]. COVID-19 information is received daily from international agencies like World Health Organization (WHO), Center for Disease Control and Prevention (CDCP) media sources including British Broadcasting Corporation (BBC), Cable News Network (CNN) among others. Both conventional and social media platforms, including WhatsApp, Twitter, and Facebook, have assisted in disseminating information with regard to the virus [4]. The National Center for Disease Control (NCDC) provides regular updates on the outbreak with support from major telecommunication operators in Nigeria including National orientation agency (NOA), Non-governmental organizations (NGOs), faith-based organizations (FBOs), and other development partners [1]. The WHO, center for disease control (CDC) and NCDC regularly publish guidelines as events unfold.

Information on COVID-19 pattern is beneficial for infection update, and stipulated prevention protocols like handwashing, physical distancing, use of facemask, as there was no cure for the coronavirus. Helplines for each state were established by the NCDC for immediate reporting of suspected cases and for possible contact tracing to prevent further spread of the disease [1]. Despite efforts to provide regular patterned and beneficial information to Nigerian residents, the majority did not believe the existence of the disease as other contradicting information circulated alongside information from reliable sources [4]. This culminated to a negative attitude among Nigerians with limited adherence to the required preventive strategies stipulated by NCDC. Also, with the introduction of lockdown policies by the government, many people became dependent on regular information sharing [5]. There was a proliferation of false news, creating anxiety, mental breakdown, panic among citizens, and even death, especially for individuals with comorbidities [6-9]. Deliberate efforts were made to debunk some myths or rumors about COVID-19 and more efforts were required to reach rural dwellers and non-literate communities [4]. From the literature reviewed, identified studies did not cover pattern and perceived benefits of COVID-19 related information in Nigeria, hence the need for this study, especially in a country where the majority of the citizens are smartphone users/social media compliant [10]. This study, therefore, assessed COVID-19 information-seeking patterns and perceived benefits among adults in Nigeria. It examined the differences in nature of the information sought by men and women in Nigeria, including if the nature of the information sought by Nigerian residents differed by location.

## Methods

**Study design:** a descriptive cross-sectional survey was used to assess patterns and perceived benefits of COVID-19 related information among adult Nigerians. This study design provides data for describing the status of phenomena or relationship with other phenomena at one specific point in time [11]. They help reveal patterns and connections that may go undetected, hence it is deemed appropriate for the present study.

**Setting:** the study was carried out in Nigeria, often referred to as the “Giant of Africa” which is made up of 36 states and its federal capital territory, Abuja. For ease of political and regional administrations, it is further divided into six geopolitical zones which include South-South, South-East, South-West, North-East, North-Central and North-West [12]. There are about 250 ethnic groups in Nigeria and the three major groups include Hausa-Fulani, Yoruba and Igbo. Although there are numerous languages in Nigeria, English is the official language of communication [13]. Nigeria was chosen for this study due to the numerous myths on COVID-19 circulating through various media handles in Nigeria during the period of this study, one of the myths includes doubting the reality of COVID-19, its prevalence and mortality rate. Hence, the authors were curious to know what the information-seeking pattern for COVID-19 was among Nigerian residents, including if they perceived any benefits for COVID-19 information.

**Sampling:** the targeted respondents included adult Nigerian residents between the ages of 18-70. Inclusion/exclusion criteria include age and ability to access social media, which was the major medium used to collect data for this study. The World Health Organization [14], recommended sample size of n = 1000, as the minimum sample for a national survey. For a 95% confidence level, a good estimate of the margin of error (or confidence interval) is given by


$$\frac{1.96x0.5}{\sqrt{N}}$$

where N is the number of participants or sample size and is the standard deviation which is unknown in this study and will be assumed to be 0.5 in this study [15]. Hence Margin of error (MoE) in this study is calculated thus (1.96x0.5/√1000) = 0.03. So, 1000 as a sample gives a margin error of less than 0.05. Therefore 1000 was considered adequate. For this study, 1039 respondents were successfully recruited. Stratified random sampling was used to recruit the sample from the population. Quota sampling was employed to ensure that the six geopolitical zones in Nigeria were represented. One state from each geopolitical zone was randomly selected using a table of random numbers which yielded the following states for the geopolitical zone, South-East (Enugu), South-South (rivers), South-West (Oyo), North-East (Bauchi), North-Central (Benue) and North-West (Sokoto). At least 100 respondents were recruited from the selected state in each geopolitical zone, and the respondents who met the criteria were included until the minimum sample size was reached.

**Data collection/procedures:** a survey tool and guidance developed by WHO prepared by Professor Betsch at the University of Erfurt, Germany, designed to guide states that wish to conduct behavioral insight studies related to COVID- 19 was adapted for this study [14]. The questionnaire was pre-validated by four independent reviewers, two experts in epidemiology and statistics, and two clinical researchers who ensured the adequacy of the items and clarity of statements before data collection. The reliability of the original instrument was established through six rounds of data collection in Germany. A pre-test of the adapted instrument was carried out to get the reliability using 10% of the sample size. One hundred (100) copies of the questionnaire were distributed to different respondents in Abia State which was not one of the selected states. Data obtained was analyzed using Guttmann´s split-half model of test internal consistency, which yielded a value 0.82 considered reliable. The responses of the pretest were not included in the data analysis, rather was used to improve the quality of the questionnaire. Due to the nature of the spread of COVID-19, an online survey was carried out. The respondents were reached through social media platforms such as WhatsApp, Facebook and Telegram. The authors ensured that there was no duplicate entry by preventing double access to respondents with the same IP address to the survey through the Google form setting. The online survey was carried out based on the checklist for reporting results of internet E-surveys (Cherries), and good practices/guidelines for conducting and reporting online research as recommended by Eysenbach [16].

**Ethical issues:** the study protocol was approved by the ethics review board, the University of Nigeria, Ituku Ozalla with a Reference No: (NHREC/05/01-2008B-FWAOOOO2458-1RB00002323). To maintain privacy and confidentiality of information, potential personal identifiers such as name, and ethnicity were excluded from the questionnaire. Also, the file containing data was password protected and access restricted to members of the research team. Participation in the study was completely voluntary, and the respondents were notified of their rights to discontinue at any stage of the study should they wish to.

**Data analysis:** data obtained was cleaned and only completed surveys were considered during data analysis. Data generated was captured and filtered in Microsoft excel 2013 and analyzed using the statistical package for social sciences (SPSS) software, version 22. Missing values were excluded. Descriptive statistics were carried out for all the variables using frequencies, percentages, mean and standard deviation. Satisfaction with information obtained was assessed on a five-point scale, a mean score of ≥2.5 was considered satisfactory while a score of ≤2.5 was considered unsatisfactory. Inferential statistics such as Chi-square were used to test if there is any difference between some of the categorical variables (gender, nature of information, and location of respondents) used in this study. A p-value of <0.05 was considered statistically significant at 95% confidence intervals.

## Results

**Sociodemographic characteristics of respondents:** the mean age is 31.0±10.20 and of 1039 respondents, 411 (49.6%) were male and 628 (60.4%) females. All except one had some level of formal education. Majority 911 (87.7%) had tertiary, 542 (52.2%) indicated to be health professionals. Based on location, around a quarter 827 (79.6%) of the respondents lived in the urban area while the remaining proportion 210 (20.2%) reside in the rural area (Table 1).

**Table 1:** sociodemographic information of the respondent (N=1039)

S/N	Items	Frequency	Percentage
1	**Age**		
	>20	38	3.7
	20-29	521	50.1
	30-39	296	28.5
	40-49	101	9.7
	50-59	64	6.2
	60-69	17	1.6
	>70	2	0.2
	Minimum	18.0	
	Maximum	73.0	
	Mean±SD	31.0±10.20	
2	**Gender**		
	Male	411	39.6
	Female	628	60.4
3	**Level of education**		
	No formal education	1	0.1
	Primary education	39	3.8
	Secondary education	88	8.5
	Tertiary	911	87.7
5	**Where do you live?**		
	Rural area	210	20.2
	Urban area	827	79.6

**Pattern of COVID-19 information sought by adult resident Nigerians:** in Table 2 which assessed intent and frequency of information sought indicates that almost half of the participants 498 (47.9%) highly desired to know about the novel virus with a mean response of 3.85±1.37. Regarding how often they sought information related to the virus, above one quarter indicated they always 311 (29.9) looked up facts about COVID-19 and almost the same percentage reported they did that sometimes 285 (27.4%). Pertaining to the nature of information sought (Table 3), participants were mostly interested in getting more information on treatment protocols 647 (62.3%). This was followed by information regarding some remedies to prevent COVID-19 infection 484 (46.6%). Statistics of spread of COVID-19 482 (46.4%), government guidelines regarding public interaction 406 (39.1%) and update on clinical trials 361 (34.7%) were other highly ranked items. Information on helplines 156 (15.0%) and guidelines on migration 209 (20.1%) were the least desired information.

**Table 2:** pattern (intent and frequency) of information on COVID-19 sought by Nigerians (N=1039)

Question	Not much	2	3	4	A lot more
To what extent do you desire to know more about COVID-19	119 (11.5)	61 (5.9)	173 (16.7)	188 (18.1)	498 (47.9)
Mean±SD	3.85±1.37				
**Frequency of seeking information on COVID-19**	**Never**	**Rarely**	**Sometimes**	**Often**	**Always**
How often do you seek for COVID-19 related information?	155 (14.9)	137 (13.2)	285 (27.4)	151 (14.5)	311 (29.9)
Mean±SD	3.3±1.40				

**Table 3:** pattern (nature) of COVID-19 information (multiple options)

Which information on COVID-19 do you seek for? I would like to get more information on	Frequency	Percentage (%)
Statistics of spread of COVID-19	482	46.4
Government guidelines regarding public interaction	406	39.1
Protocol to organizing social events	226	21.8
Protocols for school resumption	263	25.3
Guidelines on migration	209	20.1
Location of testing centres	296	28.5
Symptoms of COVID-19	237	22.8
Information on helplines	156	15.0
Updates on clinical trials	361	34.7
Home remedies to prevent COVID-19 infection	484	46.6
Treatment protocols	647	62.3

**Satisfaction with available information on COVID-19:** this was assessed as shown in Table 4 and based on the degree of satisfaction, a quarter 261 (25.1%) were highly satisfied, almost a quarter 259 (24.9%) were satisfied while above quarter were neutral 270 (26.6%). A reasonable number indicated dissatisfaction with the information to varying levels, 93 (9.0%) were very dissatisfied while 156 (15.0%) were dissatisfied. Overall, participants are satisfied with the information received regarding COVID-19 with a mean response of 3.4±1.25 where 5 is 'very satisfied´.

**Table 4:** satisfaction with available information on COVID-19

Question	Very disatisfied	Dissatisfied	Neutral	Satisfied	Very satisfied
How satisfied are you with the information available to you on COVID-19?	93 (9.0)	156 (15.0)	270 (26.0)	259 (24.9)	261 (25.1)
Mean±SD	3.4±1.25				

**Perceived benefits of COVID-19 related information:** the result showed (Table 5) that 934 (89.9%) found information on COVID-19 beneficial as it helped them understand the preventive measures 867 (83.4%), kept them informed of the necessary precautions in public places 658 (63.3%), gave them update on the infection rate, recovery rate and death rate 584 (56.4%), made them more vigilant with social distancing 471 (45.3%), provided them with information on when and how to use masks 468(45.0%) and helped them to understand the severity of the disease 400 (38.5%).

**Table 5:** perceived benefits of COVID-19 related information

Questions	Frequency	Percentage
Has information on COVID-19 been helpful or beneficial to you?		
Yes	934	89.9
No	105	10.1
If yes, in what ways?		
It helped me understand the preventive measures for the novel virus	867	83.4
It helps me get up date on the infection rate, recovery rate and death rate	584	56.4
It helped me learn when and how to use face masks	468	45.0
It kept me informed of the necessary precautions in public places	658	63.3
It helped me learn measures taken by the government to protect its citizens	379	36.5
It made me more vigilant with social distancing	471	45.3
It made me understand the severity of the disease	400	38.5
It helped me restructure my business to the current situation	263	25.3

**Chi-square test of difference in information sought by men and women in Nigeria:** from the result, there was a significant difference in seeking information on government guidelines regarding public interaction (X^2^= 4.009, p = 0.026), and home remedies to prevent COVID-19 infection (X^2^= 0.00, p = 0.013) as more women sought such information than men from the contingency table. However, there was no difference between males and females regarding information on the other items in the table 6.

**Table 6:** chi-square test of difference in nature of information sought and gender

Nature of information	Male	Female	X^2^	P-value
Statistics of spread of COVID-19	203 (42.1)	279 (57.9)	2.462	0.066
Government guidelines regarding public interaction	176 (43.3)	230 (56.7)	4.009	0.026
Protocol to organizing social events	95 (42.8)	127 (57.2)	1.329	0.141
On protocols for school resumption	108 (41.1)	155 (58.9)	0.335	0.306
Guidelines on migration	84 (40.2)	125 (59.8)	0.444	0.447
Clinical trials	78 (36.1)	138 (63.9)		
Location of testing centers	114 (38.5)	182 (61.5)	0.244	0.139
Symptoms of COVID-19	93 (39.1)	145 (60.9)	0.863	0.462
Information on helplines	67 (42.9)	89 (57.1)	0.347	0.197
Updates on clinical trials	138 (38.2)	223 (61.8)	1.354	0. 284
Home remedies to prevent COVID-19 infection	173 (35.8)	310 (64.2)	0.22	0.013
Treatment protocols	248 (38.3)	399 (61.7)	0.326	0.165

**Test (chi-square) of difference in nature of information sought based on location (urban/rural):** as shown in Table 7 more urban residents than rural sought information on statistics of spread of COVID-19 (X^2^=13.164, p =0.001), government guidelines regarding public interaction (X^2^= 8.241, p=0.016). Guidelines on migration( X^2^=11.651, p=0.003), location of testing centers (X^2^= 8.247, p=0.016), updates on clinical trials (X^2^= 17.666, p=0.001), home remedies to prevent COVID-19 infection (X^2^= 8.322, p= 0.016) and Treatment protocols (X^2^= 17.974, p = 0.001).

**Table 7:** chi-square test of difference in nature of information sought and location

Nature of information sought	Rural	Urban	X^2^	P-value
Statistics of spread of COVID-19	74 (15.4)	408 (84.6)	13.164	0.001
Government guidelines regarding public interaction	64 (15.8)	342 (84.2)	8.241	0.016
Protocol to organizing social event	35 (15.8)	187 (84.2)	4.127	0.127
On protocols for school resumption	43 (16.3)	220 (83.7)	4.004	0.135
Guidelines on migration	25 (12.0)	184 (88.0)	11.651	0.003
Clinical trials	34 (15.7)	182 (84.3)	4.337	0.114
Location of testing centres	44 (21.0)	252 (85.1)	8.247	0.016
Symptoms of COVID-19	43 (18.1)	195 (81.9)	1.509	0.470
Information on helplines	26 (16.7)	130 (83.3)	1.817	0.403
Updates on clinical trials	48 (13.3)	313 (86.7)	17.666	0.001
Home remedies to prevent COVID-19 infection	79(16.4)	404 (83.6)	8.322	0.016
Treatment protocols	107 (16.5)	540 (83.5)	17.974	0.001

## Discussion

This study sheds light on information-seeking patterns concerning the novel coronavirus among adults in a developing country, Nigeria. The existence of digital media has made it possible for people to keep up to date information about COVID-19 at their convenience [17]. Meanwhile, available facts can be questionable, especially in terms of its validity and authenticity. The areas discussed in this study are aspects of the results considered more significant by the researchers to elucidate findings. When participants in this study were asked if they desired to know more about COVID-19, almost half of them indicated they would like to know a lot more about the disease which may be attributed to the novelty of the virus and uncertainties associated with the pandemic. Corroborating this finding, Mangono *et al*. [18], in their study opined that their data showed a high demand for coronavirus information among study participants compared to other health-related information. Further validating the above findings, it was observed in this study that a reasonable proportion surfed for COVID-19 information frequently which can be explained by the curiosity to know the burden of the disease, update on the treatment/control protocols and current guidelines for business activities and travels. In the wake of the pandemic, the majority of the news headlines, magazines and blogs were focused on the pandemic, hence people were presented with COVID-19 information on all media fronts. This suggests the need to ensure that information available on these media is verified and accurate to prevent erroneous beliefs and deleterious behaviours. Also, an appreciable percentage (14.9%) reported they have never sought for COVID-19 related information which may be attributed to fear of fake news on social media, lack of internet services and infrastructures to access mostly possible among rural dwellers. Information seeking behaviour was positively associated with computer access, internet access and other infrastructures in a study carried out in Ethiopia [19].

Also, social media were indicated as the most important information channels on coronavirus [20]. However, Skarpa *et al*. [21], opined that the limited use of social media demonstrates people's awareness of the spread of fake news through it. Like the study Ebrahim *et al*. [22] reported, the majority indicated they sought COVID-19 related information on several occasions (61%). This study revealed that the majority (62.3%) of adult Nigerians were usually interested in getting more information on treatment protocols, and information regarding home remedies to prevent COVID-19. This can be traceable to the fact that there was uncertainty regarding the cure of COVID-19 amid a global outbreak, thus people will most definitely be interested in knowing what options they have when infected with COVID-19. Early effective treatment of any disease can help avert progression to more serious illness, with the additional benefit of reducing the burden on healthcare systems [23]. The desire for information relating to home remedies for COVID-19 prevention could also be related to the poor state of Nigeria health care system which seemed unprepared to respond to the pandemic. Hence, the average Nigerian can self-prescribe and self-medicate while purchasing almost over the counter drugs without any professional checks or prescribing regulations in place. During the WHO´s joint external evaluation (JEE) of International health regulations (IHR) core capacities in 2017, (an independent, collaborative multi-sectoral effort to assess a country´s capacity to prevent, detect, and respond to public health risks), Nigeria scored poorly both in prevention and response, with an average score of 1.9 across the 15 which suggested that overall, there was limited capacity to prevent biological, chemical, or radiation health risk and to respond to a sudden health risk [24]. The above findings of this study underscore the need to identify and verify various treatment options both modern and traditional, recommended for the prevention and control of the novel virus while providing accurate information on the appropriate therapeutic measures.

Furthermore, a reasonable percentage (46.4%) indicated an interest in information on the statistics of the spread of COVID-19 in Nigeria. To a large extent, Nigerians were curious regarding the reality and severity of the COVID-19 pandemic outbreak in Nigeria as opposed to the developed countries, as well as the myth that Nigerians and blacks, in general, are immune to the Coronavirus [25]. The myth evolved from the belief that the virus could not withstand the tropical climate of the country. It could be that those interested in the pattern of the disease spread in Nigeria were trying to satisfy these curiosities or otherwise. This assumption portends the possibility to disregard instructions about the pandemic or minimally adopt prescribed control or preventive measure towards the virus. A study by the research firm, SBM Intel, in all 36 states of Nigeria and Abuja, found that only 68.8% of Nigerians believe that the virus is real, 14.4 per cent of Nigerians were not sure that COVID-19 is real, while 16.7% did not believe it exists [26]. A study among medical doctors in Delta state and internally displaced persons respectively showed that their respondents´ information needs were majorly causes, symptoms, test procedures, transmission, preventive measures, vulnerable groups and isolation procedures for COVID-19 virus [27,28].

Seeking information on hotlines for COVID-19 received the least responses with a percentage response of only 15% from the respondents. What does this suggest? Could it be that the hotlines were not utilized, or that the lines when dialled were not connecting, unresponsive or unattended? Are emergency hotlines operational in Nigeria? What are the people´s first line of action in cases of emergency or are the people helpless in such circumstances? These questions require more in-depth answers but were beyond the scope of this study. Hence future research in these areas is recommended. As of May 2021, Nigeria was still hosting a one-day public hearing on National toll-free emergency number (establishment) bill, 2021 [29] Efe, [30] reported a comparative study by Statista, [31] to determine the areas people need more information on COVID-19, which covered Germany, United Kingdom, and the United States. Majority of the respondents indicated they require more information on testing for COVID-19, policies for travel, risks to the health, what to do if showing symptoms of COVID-19, shopping availability, and policies for working and schooling which agrees with the present study findings. On the contrary, Ebrahim *et al*. [22], in a study reported that participants´ priority when they sought COVID-19 related information was to identify how to apply a proper self-quarantine which portrays their worries with quarantine that demands separation from their loved ones. To further assess the respondents´ satisfaction with information on COVID-19 available to them, an appreciable percentage when summed indicated they were satisfied. Satisfaction with the available COVID-19 facts may be attributed to the availability of numerous veritable sources through which information can be accessed and verified. This study agrees with the findings of a study that assessed the degree of satisfaction with the present possibilities of obtaining COVID-19 related information among parents which revealed high satisfaction level among them with 29.4% being very satisfied while 46.4% were satisfied [22]. People satisfaction is dependent on different factors such as the number of confirmed cases and deaths, the communication strategy adopted by governments to disseminate valuable and transparent COVID-19 related information [22,32].

A large percentage of respondents found information on COVID-19 beneficial because it helped them understand the preventive measures, keep informed of the necessary precautions in public places, and stay updated on the infection, recovery and death rate. According to Okan *et al*. [33], being informed or literate about the novel virus is one of the valuable means to contain the virus and prevent its spread. A study done in China revealed that people who had reliable up-to-date information about the novel virus and how to act felt more resilient and better to handle the virus [34]. Also, WHO [35], opined that accurate dissemination of information during the current pandemic will help reduce anxiety and distress among people. This assertion was validated by Ebrahim *et al*. [22] in their study who concluded that their participants´ information-seeking behaviour had the potential to influence the severity of anxiety symptoms experienced. Similar to the study findings, numerous studies reported that the major use of COVID-19 information was to identify COVID-19 symptoms; know the isolation/quarantine procedure and practice for COVID-19 patients; treatment procedure, and the drug dosage for the treatment of COVID-19 patients and educate family members/ friends about current news on COVID-19 among others [27,28,30]. Undoubtedly, being informed about the novel virus is of immense value but the authenticity of the available information should be of great concern. Women in this study sought more information regarding government guidelines on public interaction and home remedies to prevent COVID-19 infection. This may be because caring for an ailing family member is usually the responsibility of the woman in the family. Hence, the curiosity to know about possible complementary treatments that can be used for preventing or containing the virus. Women show a higher level of worry and fear about the pandemic hence are more adept in adhering to protocols and guidelines [36]. Also, existing evidence revealed that men are often unwilling and lack the motivation to engage in health-related information compared to women [37]. However, no study has directly reported on gender and nature of the information sought, hence comparison can´t be made. Furthermore, urban residents sought more information on almost all the assessed items compared to the rural dwellers. Wide disparities in the level of technological advancement and access to infrastructures between both localities may have contributed to the present finding. Validating these claims, existing evidence showed that lack of electricity to recharge phone battery or television, poor network connectivity, high level of illiteracy, and limited information sources and funds were some of the deterrents to accessing health information among rural residents [38,39]. This study's findings agrees with the results of Efe [30], who studied rural dwellers in Delta state Nigeria, and it was found that their COVID-19 information needs range from government policies, prevention, emerging news and seeking medical help.

**Implications of the findings:** adult Nigerians desire and seek COVID-19 related information to satisfy their curiosity about the pandemic and understand how to protect themselves/others through basic actions. This suggests the need for government and health authorities to provide information that is clear, intelligible, and accessible because health literacy is crucial to mitigating the spread of the virus and its possible effect on the populace. Inequitable distribution of government infrastructures such as electricity, internet access may constrain rural dwellers from having access to reliable information on COVID-19. Hence, powers that be should provide requisite amenities to promote access and organize outreach campaigns in rural communities to sensitize them with pertinent information regarding the pandemic. Also, the dependence of the populace on various media for information on statistics, guidelines, and treatment options suggests the need for authorities to ensure credence, integrity, and veracity of information available. Individuals should verify accuracy and authenticity of information obtained from any source while double-checking with colleagues and trusted health professionals. Future research should focus on factors influencing information-seeking behaviors of Nigerians, which will provide evidence-based facts that will guide policymakers in developing appropriate measures to mitigate effects of misinformation.

**Strengths and Limitations:** this study is considered as the prime study in Nigeria assessing patterns and nature of information sought by the citizenry about the existing and unprecedented COVID-19 pandemic. Other strengths of this study were the adaptation of a WHO standardized instrument for data collection and use of quota sampling to ensure representation of each geopolitical zone in the country, which made the data amenable to generalization. However, some limitations of this study were the small sample size considering the scope of the study. Also, the mode of distribution (online-survey) of the questionnaire will not be easily accessible to some rural dwellers due to several factors, including internet coverage. Hence, care should be taken when generalization is made.

## Conclusion

Nigerians are highly desirous of information on the novel COVID-19 virus. Information on treatment protocols and home remedies for prevention/treatment of the virus were highly sought by the respondents as a result of the uncertainties regarding the cure. There is need for provision of accurate and timely information through reliable sources which will help improve health literacy, practices of the populace and help curb the spread of the COVID-19 virus.

### What is known about this topic

- *There is high demand for coronavirus information and social media is regarded as the commonest channel for information dissemination and engagement regarding COVID-19;*
- *Social media as a source of COVID-19 information dissemination have often been abused as people hide under its anonymity to spread unverified COVID-19 related messages and instigate panic amongst members of the public;*
- *Health literacy is crucial to slowing down the spread of coronavirus and mitigating its possible effects on the populace*.

### What this study adds

- *COVID-19 information is desired and often sought to satisfy curiosity and understand how to protect oneself and others through basic actions;*
- *Gender plays a role in the nature of COVID-19 information sought by individuals;*
- *Inequitable distribution of government infrastructures such as electricity, internet access may constrain rural dwellers from having access to reliable information on COVID-19*.
